# Heavy metal transfer and health risk assessment in an abandoned zinc mining–affected soil–rice system in western Thailand

**DOI:** 10.1007/s10653-026-03253-1

**Published:** 2026-05-27

**Authors:** Noppol Arunrat, Sukanya Sereenonchai, Tongchana Sagulkiatpanya, Tin Ko Oo

**Affiliations:** 1 Faculty of Environment and Resource Studies, Mahidol University, Nakhon Pathom, 73170 Thailand; 2 Institute of Nutrition, Mahidol University, Nakhon Pathom, 73170 Thailand

**Keywords:** Heavy metal contamination, Soil–rice system, Zinc mining, Human health risk assessment

## Abstract

Mining activities are a major source of heavy metal contamination in agricultural systems, posing risks to food safety and human health. This study investigated the soil–rice system in a zinc (Zn) mining–affected area of western Thailand to: (1) quantify heavy metal contamination across soil and rice compartments; (2) evaluate compartment-specific transfer factors (soil–root, root–stem, and stem–grain) to elucidate metal mobility and accumulation pathways; and (3) identify soil properties influencing metal retention and mobility. Soil, root, stem, and grain samples were collected from 20 paddy fields and analyzed for As, Cd, Cr, Cu, Mn, Pb, Zn, Hg, and Fe. The results showed that although bulk soil concentrations were within national standards, the geo-accumulation index (*I*_*geo*_) indicated extremely severe Cd and Hg contamination at all sites, with Zn also reaching extremely severe levels at several locations. In addition, consistently elevated pollution load index (*PLI*) values (> 1) indicated widespread multi-metal pollution across the entire study area. Soil organic matter, cation exchange capacity, and texture were strongly correlated with heavy metal retention and transfer (*p* < 0.05). Transfer factor analysis revealed high root accumulation of As and Hg, restricted Pb and Cd mobility, and substantial grain translocation of Cu, Zn, and Mn. Despite limited mobility, grain concentrations of As (1.16 mg kg^−1^), Cd (0.98 mg kg^−1^), Pb (4.61 mg kg^−1^), and Hg (0.08 mg kg^−1^) exceeded national food safety standards. Health risk assessment showed that both children and adults faced extremely high non-carcinogenic and carcinogenic risks from rice consumption, with hazard index and cancer risk values exceeding safe thresholds by several orders of magnitude. These findings demonstrate that the legacy of mining activities continues to threaten food safety and public health through rice-based exposure, highlighting the urgent need for targeted remediation, and routine monitoring to mitigate risks and support sustainable agriculture in mining-impacted regions.

**Supplementary Information:**

The online version contains supplementary material available at 10.1007/s10653-026-03253-1.

## Introduction

Mining activities are a major source of heavy metal pollution in aquatic and terrestrial ecosystems, including As, Cd, Pb, and Zn (Mills et al., 2017). Toxic metals released through mineral extraction processes can persist in the environment, migrate through water and soil systems, and accumulate in living organisms (Aziz et al., 2023a, 2023b; Beck et al., 2020). Numerous studies have documented the environmental impacts of mining operations, particularly in regions where these activities occur near agricultural zones. For example, research in the Jiulong section of the Yalong River Basin in China found elevated copper (Cu) concentrations in upstream water systems near mining sites, especially in areas with steep slopes and limited water convergence (Li et al., 2014). Similarly, farmland soils surrounding the Nhue River mining area in Vietnam exhibited heavy metal concentrations—especially cadmium (Cd)—that exceeded national background values and safety thresholds (González et al., 2013). In Namibia (Fry et al., 2020), reported significant contamination of soil and surface dust near a copper smelter, with arsenic (As), Cu, and lead (Pb) posing potential health risks to nearby communities. The Kouh-eZar mining area in northeastern Iran is known for its gold mining activities, which have led to heavy metal pollution, particularly elevated concentrations of Cd, chromium (Cr), Cu, Pb, and zinc (Zn) (Tahmasebi et al., 2020).

In Thailand, similar concerns have emerged due to the Zn mining at the Padaeng deposit located in Mae Sot District, Tak Province, western Thailand. For over two decades, Zn ore was extracted from this site, and irrigation water passing through the mineralized area led to Cd and Zn contamination in nearby paddy fields. The environmental and public health implications of this contamination became evident in 2003, when the International Water Management Institute (IWMI) reported elevated Cd levels in rice-based systems in Thailand. Soil Cd concentrations ranged from 0.5 to 284 mg kg⁻^1^, while rice grain Cd concentrations varied between 0.05 and 7.7 mg kg⁻^1^. Notably, more than 90% of rice grain samples exceeded the Codex maximum permissible limit of 0.2 mg kg⁻^1^ for Cd, underscoring a substantial risk to food safety and public health (Simmons et al., 2003, 2005). In response, the Thai government suspended rice cultivation in affected zones starting in 2004 and encouraged a transition to non-edible crops. Subsequent studies confirmed the potential health risks of Cd exposure among local populations (Boonprasert et al., 2011; Chaiwonga et al., 2013; Chanpiwat et al., 2019; Somporn et al., 2023; Songprasert et al., 2015) while other crop types (e.g., sugarcane, flowers) were recommended as temporary land-use solutions. These regional case studies underscore a broader global concern: the vulnerability of agricultural systems—especially rice cultivation—to heavy metal contamination. Rice (*Oryza sativa* L.), the world’s third most important cereal crop, is a staple food for more than half of the global population, median intake values of 630 g day^−1^ in South Asia and 239 g day^−1^ in Southeast Asia (Bhavadharini et al., 2020), thereby representing a major pathway for dietary exposure to heavy metals. It is also a critical source of energy, vitamins, minerals, and amino acids (Proshad et al., 2019; Zeng et al., 2009). However, rice plants are particularly prone to accumulating toxic metals such as Cd, Pb, and As, especially under flooded conditions that enhance metal solubility and plant uptake (Chandrajith et al., 2012; Zhao et al., 2010). Studies have shown that rice cultivated in contaminated soils can absorb significantly higher concentrations of these metals than rice grown in uncontaminated environments, raising serious concerns for food safety and public health (Arunrat et al., 2024; Bishwajit et al., 2014; Sharma et al., 2007). Addressing the severe health effects associated with chronic exposure to heavy metals is crucial. Pb is known to cause neurological damage, kidney dysfunction, and developmental delays, particularly in children (Boskabady et al., 2018; Ebrahimi et al., 2020; Silver et al., 2016). Cd exposure has been associated with various types of cancer and kidney damage (Buha et al., 2017; Ju et al., 2017). Hg primarily affects the central nervous system and cardiovascular health (Kim et al., 2016). As, a toxic metalloid, can cause a wide range of disorders across multiple organ systems, even at low concentrations (Abdul et al., 2015; Tchounwou et al., 2012). Cr poses risks to the respiratory system and skin (Caglieri et al., 2006). Manganese (Mn) exposure may lead to adverse neurological, reproductive, and respiratory effects (Aschner et al., 2005). Zn toxicity manifests as nausea, vomiting, epigastric pain, lethargy, and fatigue when intake levels are excessively high (Fosmire, 1990). Cu overload can result in Wilson’s Disease, causing liver and brain toxicity (Chen et al., 2022). Iron (Fe) accumulation may lead to hemochromatosis, a genetic disorder characterized by iron overload (Andrews, 2000). Thus, heavy metals are of particular concern due to their persistence, bioaccumulation, and toxicity, and their co-occurrence as mixtures can result in additive or synergistic effects that amplify ecological and human health risks (Canlı et al., 2023; Çelebi et al., 2024; Güzel et al., 2022).

While previous studies have confirmed the presence of heavy metals in soils and rice cultivated near mining areas, important mechanistic and interpretative gaps remain. In particular, the influence of soil physicochemical properties—such as pH, texture, organic matter, and nutrient status—on metal mobility and bioavailability within the soil–rice continuum is still not well resolved. Moreover, most studies rely on total metal concentrations and provide limited insight into the stepwise transfer processes governing metal movement across soil–root, root–stem, and stem–grain compartments. As a result, a consistent framework for interpreting transfer factors (TFs) and identifying key accumulation pathways is lacking. To address these gaps, this study aims to: (1) quantify heavy metal contamination across soil and rice compartments in Zn mining-affected systems using inductively coupled plasma–optical emission spectrometry (ICP–OES); (2) evaluate compartment-specific transfer factors (soil–root, root–stem, and stem–grain) to elucidate metal mobility and identify critical accumulation pathways using transfer factor index and human health risk assessment; and (3) determine how soil physicochemical properties regulate these transfer processes using Pearson correlation analysis. By explicitly linking transfer dynamics with dietary exposure, this study provides new evidence that soil quality standards alone may be insufficient to protect human health in rice-based agroecosystems of Southeast Asia, and proposes a more mechanistic basis for risk assessment.

## Materials and methods

### Study areas

**Fig. 1:**
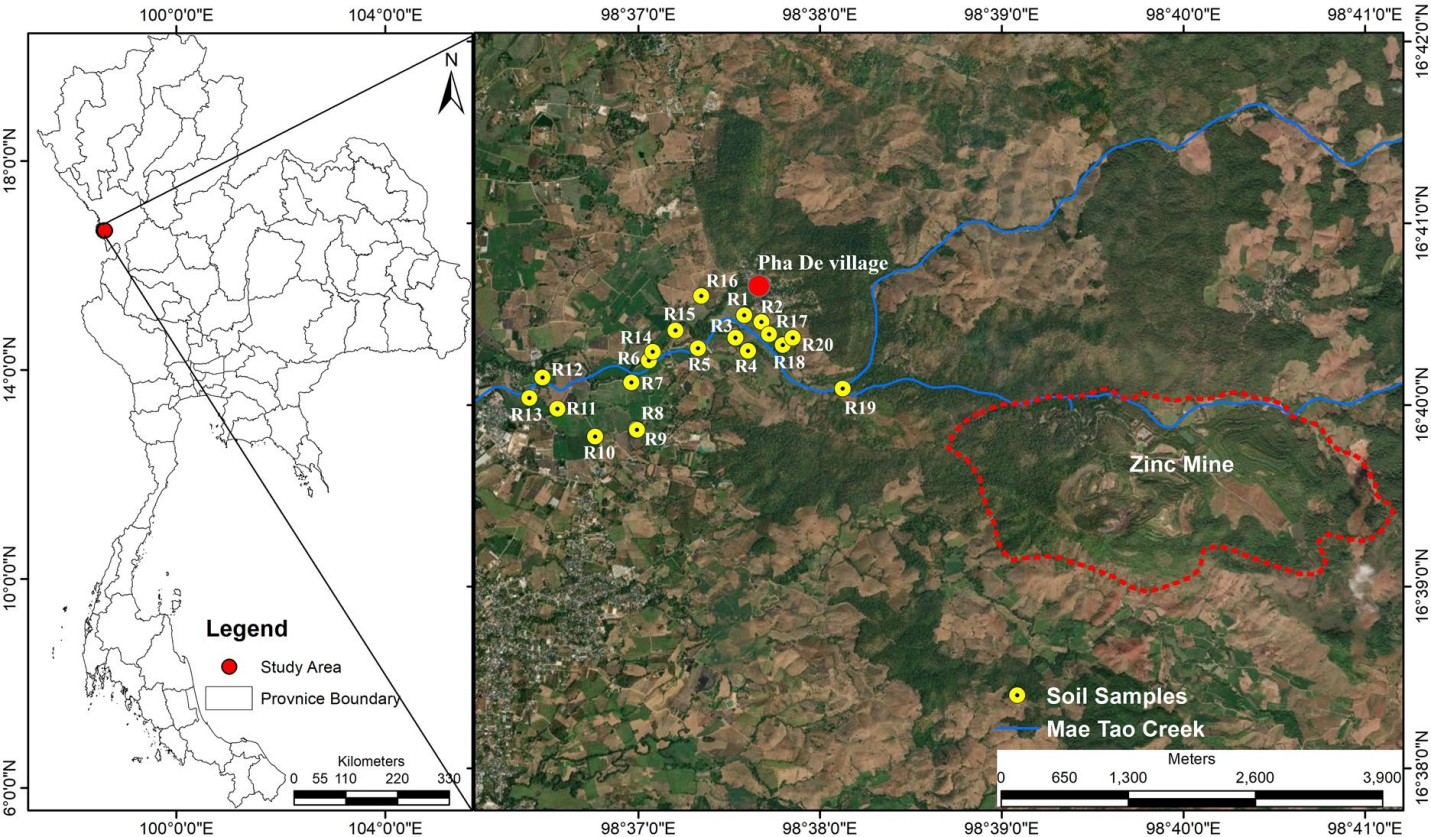
Study area

The study area is located in Pha De Village (Fig. 1), Phratat Phadaeng Subdistrict, Mae Sot District, Tak Province, western Thailand. Pha De Village was selected because it is the closest settlement to the mining area and is situated on the alluvial fan of the Mae Tao Creek, where the highest concentrations of heavy metals have been reported (Prasad et al., 2015). Zinc mining activities were initiated in 1982 with the primary objective of producing Zn metal and Zn alloys. The mine had an average annual production of 67,000 tons of zinc metal and 101,100 tons of zinc alloys (Prasad et al., 2015). Mae Tao Creek originates in the mountains of northwestern Thailand and flows through the Zn mine, Pha De and Mae Tao Mai villages, and the Mae Sot city area, before eventually discharging into the Moei River, which forms the border between Thailand and Myanmar.

The geology of the Zn deposit belongs to the Upper Triassic–Jurassic age and consists of dark grey limestone and light grey bedded limestone containing ammonites, brachiopods, and coral reefs, interbedded with calcareous shale, sandstone, and grey to brownish-red lime conglomerate (Akkajit & Tongcumpou, 2010; Reynolds et al., 2003) The ore extracted from this mine was primarily composed of zinc silicate [hemimorphite, Zn_4_(Si_2_O_7_)(OH)_2_·2H_2_O], with smaller amounts of zinc carbonate (smithsonite, ZnCO_3_) conglomerate (Akkajit & Tongcumpou, 2010; Sriprachote et al., 2012). Owing to the surrounding mountainous terrain, Tak Province experiences pronounced seasonal temperature variation, with hot summers and cool winters. Summer typically begins around mid-February and lasts until mid-May, with an average maximum temperature of 35.2 °C. The rainy season occurs from mid-May to mid-October, with an average total rainfall of 233.0 mm. Winter starts around mid-October and continues until mid-February, with an average minimum temperature of 18.0 °C. Annual rainfall ranges from 651.10 mm (74 rainy days) to 1,556.30 mm (154 rainy days). (Mae Sot District Agricultural Office, 2023).

Twenty paddy fields along Mae Tao Creek in Pha De village were investigated (Fig. 1). All fields practice rice cultivation once a year using the transplanting method. The growing season extends from May to November, during which farmers typically cultivate the ‘Khao Dawk Mali 105’ (KDML 105) rice variety. Chemical fertilizers (N–P–K) were applied at approximate rates of 156.3 kg ha^–1^ (16–20–0), 125.0 kg ha^–1^ (16–16–8), and 62.5 kg ha^–1^ (46–0–0) per growing season.

### Experimental design and sampling

Three transect lines were established within each paddy field to capture spatial variability across the field. The transects were laid out parallel to the main irrigation flow and spaced approximately 10–15 m apart. Along each transect, three sampling plots (1 m × 1 m each) were established at regular 5 m intervals. Within each plot, rice plants and soil were collected from the same location to ensure consistent representation of the soil–plant system. Soil samples were collected from a depth of 0–10 cm using a stainless-steel auger after removing surface litter, as this layer represents the most biologically active and nutrient-enriched zone where root growth and metal uptake predominantly occur. The collected soil, root, stem, and grain samples from the three plots within each transect were composited to form one representative sample per sample type (soil, root, stem, and grain). All samples were collected in December 2024 and stored in plastic bags, with approximately 2 kg per soil sample and 1 kg each for rice root, stem, and grain samples.

For geochemical background determination, soil samples were collected at a depth of 200 cm from two community forests in Phra That Subdistrict, Mae Ramat District, and Khirirat Subdistrict, Phop Phra District, Tak Province.To represent the natural geochemical background, we assumed that the deep soil layer reflects parent material derived from bedrock through long-term weathering processes and has not been influenced by anthropogenic activities.

### Soil physicochemical properties analysis

Soil samples were air-dried, ground, and passed through a 2-mm sieve prior to analysis. Soil texture was assessed using the hydrometer method (Burt, 2004). Soil pH was measured in a 1:1 soil-to-water suspension with a pH meter (Burt, 2004), while electrical conductivity (ECe) was determined from saturation paste extracts using an EC meter (Soil Survey Staff, 2014). Exchangeable calcium (Ca), magnesium (Mg), and potassium (K) were extracted with 1 M ammonium acetate at pH 7.0 and quantified using atomic absorption spectrometry (Jones, 2001; Thomas, 1982). Available phosphorus (P) was measured using the molybdate blue method following Bray II extraction (Bray & Kurtz, 1945; Jones, 2001). Soil organic carbon (SOC) was determined by potassium dichromate (K_2_Cr_2_O_7_) oxidation in sulfuric acid (Jones, 2001; Walkley & Black, 1934), and soil organic matter (SOM) was estimated by multiplying SOC by a factor of 1.5 (Shamrikova et al., 2022).

### Analysis of heavy metal concentrations

Following a conservative screening-level approach to identify potential human health concerns under worst-case exposure scenarios, the concentrations of As, Cd, Cr, Cu, Mn, Pb, Zn, Hg, and Fe were determined in soil and rice (root, stem, and grain) samples using U.S. Environmental Protection Agency Method 3051A (USEPA, 2007). Soil samples were air-dried, gently disaggregated, and thoroughly mixed to ensure homogenization, then passed through a 0.05 mm sieve. Rice root, stem, and grain samples were carefully washed with tap water followed by deionized water to remove adhering soil particles and dust before being oven-dried at 60 °C to a constant weight. Dried soil and plant samples were ground and sieved to a uniform powder. A 0.5 g subsample was digested with 10 mL of concentrated nitric acid (HNO_3_) using a high-performance microwave digestion system (Milestone, Ethos Easy, Italy) at 175 °C for 40 min. The digested solutions were filtered and analyzed for heavy metal concentrations using inductively coupled plasma–optical emission spectrometry (ICP–OES 5800; Agilent Technologies, USA).

### Quality assurance and quality control (QA/QC)

All calibration standards were prepared within the concentration ranges reported in the literature for ICP-OES analysis. The limit of detection (LOD) for ICP-OES analysis was determined based on the standard deviation of the blank signal, calculated as three times the standard deviation of the blank. The LOD values for each element were as follows: As: 5.0 µg L^−1^, Cd: 0.3 µg L^−1^, Cr: 0.5 µg L^−1^, Cu: 0.3 µg L^−1^, Mn: 0.09 µg L^−1^, Pb: 5.3 µg L^−1^, Zn: 0.2 µg L^−1^, Hg: 0.3 µg L^−1^, and Fe: 0.4 µg L^−1^. The limit of quantification (LOQ) was calculated using the σ–slope method. Accordingly, the LOQ values (µg L⁻^1^) were as follows: As (16.7), Cd (1.0), Cr (1.7), Cu (1.0), Mn (0.30), Pb (17.7), Zn (0.67), Hg (1.0), and Fe (1.33). A linear calibration curve was established using a least-squares regression approach, yielding a correlation coefficient (R^2^) of ≥ 0.995. To ensure analytical accuracy, quality control measures included the repeated analysis of a known standard after every 10 samples. Each sample was analyzed in duplicate, with recovery rates ranging from 93 to 105% and relative standard deviations below 10%, confirming the reliability and precision of the measurements.

### Heavy metal absorption and transferability

#### Geo-accumulation

The geo-accumulation index (*I*_*geo*_) is a widely used metric for assessing the degree of heavy metal enrichment in soil relative to background or baseline concentrations (Abrahim & Parker, 2008). The *I*_*geo*_ was calculated using Müller’s equation (Muller, 1969), as follows:

1 $$I_{geo} = log_{2} \left[ {\frac{{C_{n} }}{{1.5 \times B_{n} }}} \right]$$

where, *Cn* represents the concentration of the heavy metal in the soil sample (mg kg⁻^1^), and *Bn* denotes the geochemical background value of the element “n”. The background values in this study were the concentrations of As, Hg, Cd, Cr, Pb, Cu, Mn, Zn, and Fe, which were 21.67, 0.0003, 0.0003, 16.67, 9.33, 48.84, 302.67, 15.34, and 12,883.50 mg kg⁻^1^, respectively. A constant factor of 1.5 is applied as the background matrix correction factor to account for natural variations due to lithogenic effects (Muller, 1969). The classification of *I*_*geo*_ values is as follows: *I*_*geo*_ ≤ 0 is no contamination, 0 < *I*_*geo*_ ≤ 1 is light to moderate, 1 < *I*_*geo*_ ≤ 2 is moderate, 2 < *I*_*geo*_ ≤ 3 is moderate to heavy, 3 < *I*_*geo*_ ≤ 4 is heavy, 4 < *I*_*geo*_ ≤ 5 is heavy to extremely serious and *I*_*geo*_ ≥ 5 is extremely serious, respectively.

#### Pollution load index

The pollution load index (*PLI*) was employed to assess the contamination status of soils with respect to heavy metals (Negahban & Mokarram, 2021). The *PLI* is calculated as the nth root of the product of the contamination factors (*CF*) for *n* different metals across various sites (Tomlinson et al., 1980). This index provides a straightforward means of quantifying the overall level of heavy metal accumulation resulting from soil degradation.

2 $$PLI = \sqrt[n]{{CF_{1} \times CF_{2} \times CF_{3} \times \ldots \times CF_{n} }}$$

3 $$CF = \frac{{C_{n} }}{{B_{n} }}$$

where, *CF* is the metal representing the contamination factor of each metal, and *n* denotes the number of analyzed metals. The interpretation of *PLI* values is as follows: *PLI* < 1 indicates no pollution; *PLI* = 1 suggests baseline levels of contaminants; and *PLI* > 1 signifies pollution.

### Translocation Factor

The transfer factor (*TF*) is an index used to assess the mobility and uptake of heavy metals from soil to plant tissues—including roots, stems, and grains—and was calculated as follows (Lorestani et al., 2011):

4 $$TF_{soil - root} = \frac{{C_{root} }}{{C_{soil} }}$$

5 $$TF_{root - stem} = \frac{{C_{stem} }}{{C_{root} }}$$

6 $$TF_{stem - grain} = \frac{{C_{grain} }}{{C_{stem} }}$$

where *TF*_*soil-root*_, *TF*_*root-stem*_, and *TF*_*stem-grain*_ represent the transfer factors from soil to root, root to stem, and stem to grain, respectively. *C*_*soil*_, *C*_*root*_, *C*_*stem*_, and *C*_*grain*_ denote the concentrations of heavy metals in the soil, root, stem, and grain, respectively. A *TF*_*soil-root*_ > 1.00 indicates the plant's potential to hyperaccumulate metals, absorbing more than what is available in the soil. A value ≤ 1.00 implies proportional uptake without hyperaccumulation (Yan et al., 2020). A *TF*_*root-stem*_ > 1.00 signifies high internal mobility of metals and potential accumulation in the stem, whereas ≤ 1.00 suggests restricted translocation. The *TF*_*stem-grain*_ is especially important for food safety; values > 1.00 indicate significant accumulation in edible parts, posing potential health risks, while values ≤ 1.00 suggest limited translocation and lower contamination risk.

### Human health risk assessment

The population sampling focused on local residents of Pha De Village, where soil, rice root, stem, and grain samples were collected from twenty paddy fields. The surveyed population was divided into two age groups: children (6–17 years) and adults (18 years and older) (Department of Health, 2017). In total, 117 local residents participated in the household survey, including 34 children and 83 adults, representing all households associated with the twenty paddy fields studied. Information collected included age, body weight, and daily rice consumption. Body weight was measured using a weighing scale, while age and daily rice consumption were obtained from a questionnaire. Data for children were provided by their parents, whereas adults completed the questionnaire themselves.

The assessment of heavy metal contamination and related health risks—both cancerous and non-cancerous—from consuming contaminated crops was conducted by calculating hazard quotients (HQs). These HQs were then added together to derive the hazard index (HI).

#### Average daily dose

The average daily dose (ADD) was used to quantify oral exposure to heavy metal contaminants through rice consumption over time, expressed as the daily intake per unit of body weight (mg kg⁻^1^ day⁻^1^) (Arunrat et al., 2024). It was calculated using the following equation:

7 $$ADD = \frac{C \times IR \times EF \times ED}{{BW \times AT}}$$

where *C* represents the concentration of heavy metals in rice grain (mg kg⁻^1^); *IR* is the average daily intake of rice grain (0.23 ± 0.012 kg day⁻^1^ for adults and 0.11 ± 0.011 kg day⁻^1^ for children); *BW* is the body weight (68.3 ± 6.5 kg for adults and 41.2 ± 7.1 kg for children); *EF* is the exposure frequency (365 days year⁻^1^); *ED* is the exposure duration (77 years, representing the average human lifespan); and *AT* is the average time, calculated as the product of EF and ED (days).

#### Non-carcinogenic risk

The reference dose (RfD), representing the maximum acceptable oral intake of a toxic element, was adopted from the United States Environmental Protection Agency (USEPA). Accordingly, the hazard quotient (HQ) was calculated using the following equation (Yan et al., 2020):

8 $$HQ = \frac{ADD}{{RfD}}$$

where, ADD represents the average daily dose, and RfD is the reference dose (see Supplementary Material, Table S1). An HQ value less than 1 indicates no potential non-carcinogenic risk, whereas an HQ value greater than 1 suggests potential non-carcinogenic risk.

The hazard index (HI) was calculated by summing the hazard quotients (HQs) of all heavy metals to evaluate the potential health risks associated with combined exposure (Aziz et al., 2023a, 2023b).

9 $$HI = \sum HQ$$

An HI value less than 1.00 indicates no potential non-carcinogenic health risk, whereas an HI greater than 1.00 suggests a possible non-carcinogenic health risk.

#### Carcinogenic risk

Cancer risk (CR) was estimated by multiplying the average daily intake of a specific heavy metal over a lifetime by its cancer slope factor (CSF), which reflects the metal's carcinogenic potency (see Supplementary Material, Table S1). CR represents the incremental probability that an individual will develop cancer over their lifetime as a result of exposure to that heavy metal (Yan et al., 2020).

10 $$CR = ADD \times CSF$$

The total cancer risk (CRₜₒₜₐₗ) was used to evaluate the combined effects of multiple carcinogenic elements and was calculated by summing the individual cancer risks (CR) associated with each element (Aziz et al., 2023a, 2023b).

11 $$CR_{total} = \sum CR$$

Cancer risks ranging from 1.0 × 10^−6^ to 1.0 × 10^−4^ are generally considered acceptable (Fan et al., 2017), whereas values exceeding 1.0 × 10^−4^ suggest a high potential for carcinogenic health risk.

### Statistical analysis

Statistical analyses were conducted using IBM SPSS statistics software (Version 20, IBM Corporation, USA). One-way ANOVA was performed to assess significant differences in heavy metal concentrations in soil, root, stem, and grain. The *TFs* and pollution indices showed highly skewed distributions spanning several orders of magnitude. Therefore, log₁₀ transformation was applied to *PLI*, *TF*_*soil-root*_, *TF*_*root-stem*_, and *TF*_*stem-grain*_ values prior to statistical comparison, visualization, and interpretation to stabilize variance and reduce the influence of extreme values. Raw (non-transformed) values were retained for health risk calculations to maintain consistency with established USEPA risk assessment protocols. Subsequently, Tukey’s honestly significant difference (HSD) test was employed for multiple post hoc mean comparisons when the ANOVA result was significant at *p* ≤ 0.05. Pearson correlation analysis was used to examine linear relationships between soil physicochemical properties and heavy metal concentrations, assuming approximate normality of averaged site-level data. Prior to analysis, variables exhibiting strong multicollinearity (variance inflation factor, VIF > 10) or high intercorrelation (|r|> 0.7) were excluded to ensure statistical robustness (Cao et al., 2015; Praveena & Omar, 2017). This exploratory approach was intended to identify dominant soil factors influencing metal retention and mobility rather than to develop predictive models.

## Results

### Soil physicochemical properties

Soil pH across the study area was predominantly neutral to slightly alkaline, ranging from 6.0 to 7.9. The majority of sites (19 out of 20) exhibited a pH of 7.0 or higher. Site R9 was the only exception, with a slightly acidic mean pH of 6.0. The highest pH value was recorded at 7.9 (Table 1). ECe values were consistently low across all sites, ranging from 0.2 to 0.5 dS m^−1^, indicating that the soils are generally non-saline (Table 1). SOM was moderate, with site averages ranging from 2.0% to 4.4% (Table 1). CEC is an important indicator of soil fertility, varied considerably, from 11.8 to 27.1 cmol kg^−1^. This broad range reflects differences in the soils’ capacity to retain essential nutrients (Table 1). Total N was generally low and consistent across all sites, with values of either 0.1% or 0.2% (Table 1). Available macronutrient concentrations varied widely among the sites. Available P ranged from 22.0 to 99.0 mg kg^−1^, suggesting a broad spectrum of P availability. Available K levels ranged from 36.0 to 95.7 mg kg^−1^. Available Ca and available Mg concentrations were generally high, in line with the neutral to alkaline soil pH. Available Ca was the most abundant exchangeable cation, with values ranging from 1,145.3 to 4,969.3 mg kg^−1^. Available Mg levels varied between 141.0 and 316.7 mg kg^−1^. Soil texture varied across sites (Table 1). Sand content ranged from 17.3 to 48.0%, silt from 19.7 to 53.0%, and clay from 24.7 to 45.7%. Textural classifications were diverse, but many soils were fine-textured, including loams, clay loams, and clays (Table 1).

**Table 1:** Soil physicochemical properties across the studied sites (Mean ± Standard Deviation)

Sites	pH	EC (ds m^−1^)	SOM (%)	CEC (cmol kg^−1^)	Total N (%)	Avali. P (mg kg^−1^)	Avali. K (mg kg^−1^)	Avali. Ca (mg kg^−1^)	Avali. Mg (mg kg^−1^)	% Sand	% Silt	% Clay
R1	7.4 ± 0.38	0.3 ± 0.16	3.0 ± 0.17	16.4 ± 1.15	0.13 ± 0.02	68.0 ± 12.17	65.3 ± 16.44	3308.0 ± 959.03	249.7 ± 23.01	47.3 ± 6.11	24.3 ± 4.04	28.3 ± 2.08
R2	7.6 ± 0.49	0.2 ± 0.07	3.1 ± 0.52	19.8 ± 4.58	0.13 ± 0.03	56.3 ± 12.10	79.7 ± 37.02	4143.3 ± 1720.90	316.7 ± 82.12	37.3 ± 19.01	25.3 ± 7.09	37.3 ± 12.01
R3	7.9 ± 0.15	0.2 ± 0.06	2.7 ± 1.42	20.0 ± 7.85	0.13 ± 0.07	37.7 ± 21.22	80.3 ± 42.03	4969.3 ± 847.77	310.7 ± 188.11	42.0 ± 24.25	19.7 ± 8.08	38.3 ± 21.39
R4	7.6 ± 0.21	0.4 ± 0.15	2.8 ± 1.33	21.4 ± 5.39	0.14 ± 0.08	22.7 ± 6.11	57.7 ± 17.21	3035.0 ± 829.99	270.7 ± 123.00	43.3 ± 26.41	23.3 ± 11.50	33.3 ± 15.31
R5	7.3 ± 0.36	0.3 ± 0.03	3.1 ± 0.53	16.8 ± 2.40	0.15 ± 0.03	51.3 ± 34.96	36.0 ± 8.54	1721.7 ± 177.66	244.3 ± 34.00	48.0 ± 0.00	24.3 ± 3.06	27.7 ± 3.06
R6	7.6 ± 0.26	0.3 ± 0.10	3.8 ± 0.55	23.3 ± 2.59	0.17 ± 0.02	81.7 ± 17.10	95.7 ± 47.65	2328.0 ± 629.99	287.7 ± 70.47	22.7 ± 3.06	31.7 ± 6.11	45.7 ± 4.16
R7	7.8 ± 0.10	0.3 ± 0.07	4.0 ± 0.67	21.4 ± 2.83	0.19 ± 0.03	59.7 ± 29.87	72.3 ± 9.07	3964.3 ± 828.37	214.0 ± 23.52	17.3 ± 1.15	41.7 ± 10.26	41.0 ± 10.58
R8	7.0 ± 0.56	0.2 ± 0.02	2.7 ± 0.15	13.9 ± 0.92	0.12 ± 0.02	99.0 ± 57.00	41.7 ± 10.69	1444.7 ± 178.89	171.3 ± 4.04	35.0 ± 11.36	36.7 ± 10.69	28.3 ± 2.31
R9	6.0 ± 0.59	0.2 ± 0.11	2.9 ± 0.32	11.8 ± 1.24	0.13 ± 0.02	86.7 ± 33.13	42.0 ± 12.49	1145.3 ± 273.23	141.0 ± 44.03	30.7 ± 9.87	35.7 ± 3.06	33.7 ± 7.57
R10	6.9 ± 0.92	0.5 ± 0.22	2.4 ± 1.04	15.2 ± 7.74	0.12 ± 0.05	58.0 ± 3.00	38.3 ± 14.22	1757.0 ± 408.19	194.0 ± 44.51	36.0 ± 27.78	28.7 ± 10.50	35.3 ± 19.76
R11	7.8 ± 0.12	0.3 ± 0.12	4.1 ± 0.76	22.2 ± 1.53	0.17 ± 0.02	54.0 ± 17.35	64.7 ± 15.50	3018.3 ± 562.00	201.7 ± 28.59	24.0 ± 6.00	38.3 ± 11.37	37.7 ± 14.19
R12	7.7 ± 0.06	0.5 ± 0.15	3.8 ± 0.38	20.1 ± 5.27	0.17 ± 0.02	48.0 ± 5.29	62.0 ± 11.14	3387.3 ± 336.39	198.7 ± 17.95	25.7 ± 7.09	37.0 ± 6.56	37.3 ± 11.93
R13	7.2 ± 0.23	0.3 ± 0.11	3.6 ± 0.15	21.9 ± 3.01	0.17 ± 0.01	35.3 ± 14.47	55.3 ± 8.50	2243.7 ± 403.76	209.3 ± 36.36	24.7 ± 6.35	35.3 ± 5.51	40.0 ± 10.00
R14	7.4 ± 0.35	0.3 ± 0.07	3.8 ± 0.78	25.7 ± 2.89	0.16 ± 0.02	25.3 ± 6.11	54.3 ± 14.01	2913.0 ± 777.30	253.3 ± 34.21	21.0 ± 3.46	36.3 ± 16.17	42.7 ± 16.04
R15	7.7 ± 0.10	0.3 ± 0.06	3.6 ± 0.64	21.8 ± 3.87	0.15 ± 0.03	27.3 ± 4.04	59.0 ± 6.24	2903.7 ± 259.79	182.3 ± 31.88	22.3 ± 4.16	53.0 ± 11.14	24.7 ± 13.32
R16	7.2 ± 0.62	0.2 ± 0.06	4.4 ± 0.17	27.1 ± 0.71	0.19 ± 0.01	63.3 ± 17.39	79.7 ± 15.89	2333.0 ± 430.97	308.3 ± 27.06	21.7 ± 6.43	45.0 ± 11.14	33.3 ± 11.02
R17	7.6 ± 0.06	0.2 ± 0.09	3.6 ± 0.17	19.0 ± 0.40	0.16 ± 0.02	63.3 ± 22.05	46.3 ± 11.59	2301.3 ± 562.41	231.7 ± 66.15	29.7 ± 4.62	27.0 ± 13.11	43.3 ± 9.87
R18	7.7 ± 0.00	0.5 ± 0.11	3.9 ± 0.58	16.5 ± 3.68	0.16 ± 0.03	27.0 ± 2.00	42.0 ± 13.00	2960.7 ± 724.55	189.7 ± 49.22	32.3 ± 17.47	27.3 ± 9.29	40.3 ± 9.61
R19	7.9 ± 0.06	0.2 ± 0.06	2.0 ± 0.85	14.2 ± 2.29	0.10 ± 0.04	25.3 ± 9.45	37.0 ± 17.06	2245.7 ± 798.74	189.0 ± 58.39	27.7 ± 16.04	38.7 ± 8.39	33.7 ± 9.81
R20	7.4 ± 0.55	0.2 ± 0.11	3.1 ± 0.82	16.0 ± 5.00	0.16 ± 0.04	55.7 ± 41.04	59.3 ± 6.43	1937.0 ± 471.51	146.0 ± 27.78	27.0 ± 9.17	41.0 ± 3.46	32.0 ± 6.00

### Heavy metal concentrations in soil and plant compartments

Heavy metal concentrations in soil and plant compartments compared with their respective risk control values are presented in Table 2. All heavy metal concentrations measured in the soil complied with the national standard limits set by the Pollution Control Department, Thailand (2021), with no exceedances detected. Fe showed the highest accumulation in the root (18,401.58 mg kg^−1^), which was substantially higher than in the soil (265.99 mg kg^−1^), stem (114.52 mg kg^−1^), and grain (67.89 mg kg^−1^). Mn and Zn also exhibited high concentrations in the soil (620.89 and 360.62 mg kg⁻^1^, respectively), followed by roots and stems, and were lowest in the grain. It is important to note that both Mn and Zn are essential micronutrients required for plant growth and human health; however, excessive accumulation may still pose potential risks. The root compartment had elevated levels of several metals, particularly As at 41.59 mg kg⁻^1^ and Cd at 6.82 mg kg^−1^, both higher than corresponding soil concentrations. In the grain, although metal concentrations were generally the lowest among compartments, several metals exceeded the maximum allowable limits set by the Ministry of Public Health, Thailand (2020). Specifically, As (1.16 mg kg^−1^), Cd (0.98 mg kg^−1^), Pb (4.61 mg kg^−1^), and Hg (0.08 mg kg^−1^) all surpassed their respective food safety thresholds of 0.2, 0.4, 0.2, and 0.02 mg kg^−1^, raising concerns over potential health risks from consumption. Hg concentrations, though numerically low, exceeded the grain safety standard across all plant parts. Overall, the results highlight substantial metal uptake by plant tissues, particularly roots, and the potential transfer of toxic metals to edible grain portions above acceptable limits.

**Table 2:** Heavy metal concentrations (mg kg^−1^) in soil, root, stem, and grain (Mean ± Standard Deviation)

Compartment	As	Cd	Cr	Cu	Mn	Pb	Zn	Hg	Fe
Soil	21.53 ± 8.59^a^	8.28 ± 8.62^a^	21.57 ± 7.43^a^	17.20 ± 6.22^a^	620.89 ± 273.33^a^	36.88 ± 16.03^a^	360.62 ± 319.26^a^	0.12 ± 0.05^a^	265.99 ± 115.52^a^
Root	41.59 ± 38.01^b^	6.82 ± 6.78^b^	5.88 ± 4.32^b^	9.32 ± 6.63^b^	374.18 ± 329.08^b^	14.88 ± 13.59^b^	219.98 ± 184.83^b^	0.17 ± 0.07^b^	18,401.58 ± 5573.30^b^
Stem	23.92 ± 28.92^c^	2.58 ± 2.03^c^	4.18 ± 4.45^c^	7.82 ± 7.88^c^	249.18 ± 217.54^c^	6.07 ± 8.23^c^	91.48 ± 58.79^c^	0.11 ± 0.07^a^	114.52 ± 73.10^c^
Grain	1.16 ± 0.60^d^	0.98 ± 0.71^d^	0.34 ± 0.41^d^	4.01 ± 7.11^d^	99.89 ± 98.17^d^	4.61 ± 7.54^d^	34.88 ± 19.19^d^	0.08 ± 0.06^a^	67.89 ± 13.17^d^
Risk control values									
Soil^*^	≤ 25	≤ 762	≤ 212	≤ 35,040	≤ 19,640	≤ 800	–	≤ 263	–
Grain^**^	≤ 0.2	≤ 0.4	–	–	–	≤ 0.2	–	≤ 0.02	–

^a^^–d^ denote significant statistical differences (*p* < 0.05) among the compartments. Sources: * Pollution Control Department (2021), **Ministry of Public Health (2020)

**Fig. 2:**
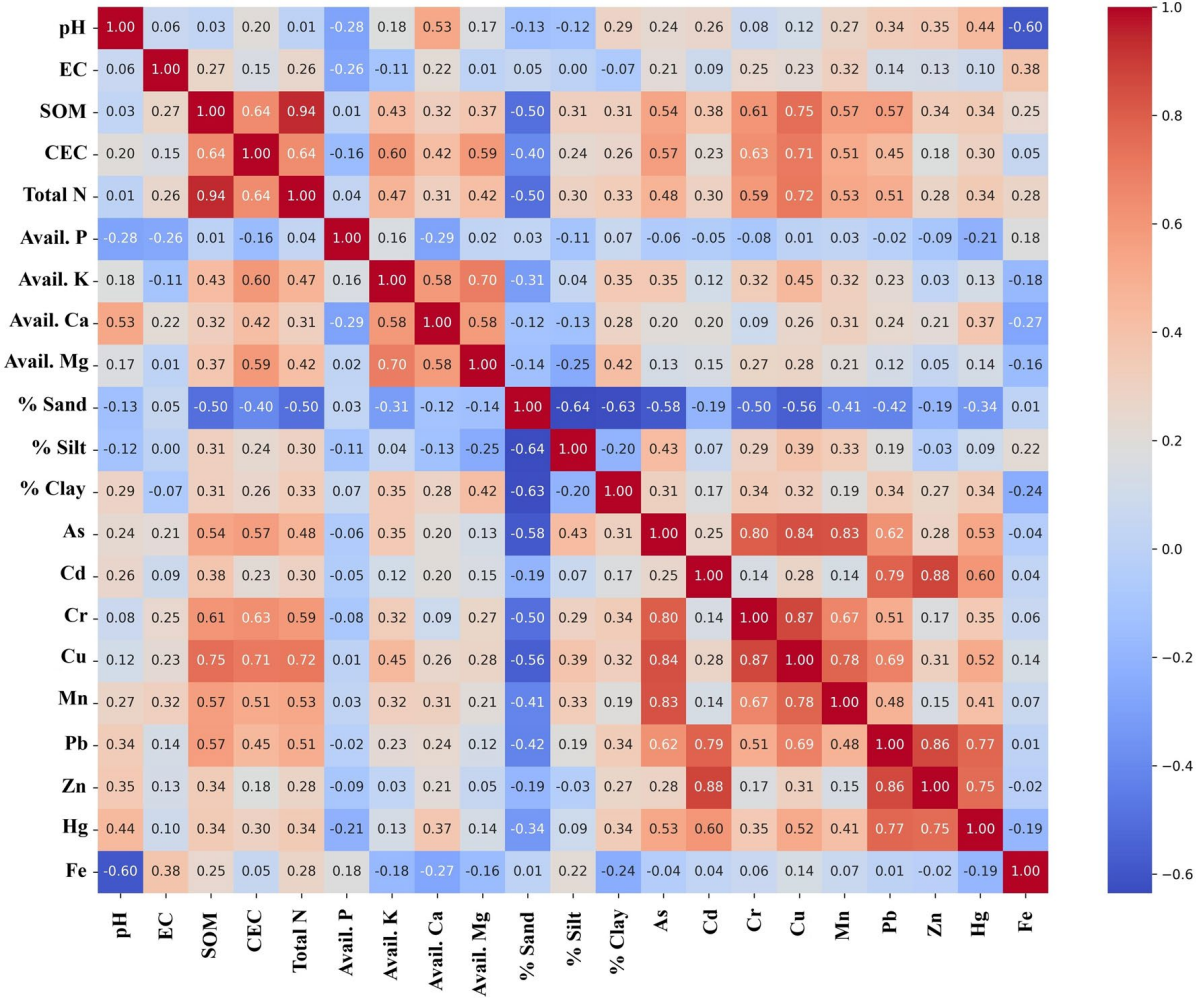
Pearson correlation matrix showing relationships among soil properties and soil heavy metal concentrations (n = 60). Only significant correlations with *r* > 0.5 are considered. The color scale represents the strength and direction of the correlations, with red indicating positive and blue indicating negative relationships

Among soil properties, SOM exhibited a very strong positive correlation with Total N (*r* = 0.94), highlighting its critical role as the primary nitrogen reservoir in these soils. SOM also showed a strong positive correlation with CEC (*r* = 0.64), reinforcing its importance in enhancing the soil’s nutrient retention capacity. In turn, CEC demonstrated strong positive associations with avail. K (*r* = 0.60) and avail. Mg (*r* = 0.59). In contrast, a notable negative correlation was observed between pH and Fe concentrations (*r* = − 0.60), indicating that iron availability tends to decrease with increasing pH (Fig. 2). Regarding soil texture, sand content showed significant negative correlations with several key soil and metal parameters. Specifically, sand was negatively correlated with SOM (*r* = − 0.50), Total N (*r* = − 0.50), CEC (*r* = − 0.40), Cu (*r* = − 0.56), and As (*r* = − 0.58), suggesting that sandy soils are less effective at retaining SOM, nutrients, and certain trace metals. Conversely, clay content was moderately positively correlated with avail. Mg (*r* = 0.42) and Cu (*r* = 0.32), indicating enhanced metal retention in finer-textured soils (Fig. 2). The inter-elemental analysis of heavy metals revealed strong positive correlations among several metal pairs, suggesting shared sources or similar geochemical behavior. Cu exhibited very strong correlations with Cr (*r* = 0.87) and As (*r* = 0.84). Likewise, As was strongly correlated with Cr (*r* = 0.80) and Mn (*r* = 0.83). Cd was strongly associated with Zn (*r* = 0.88) and Pb (*r* = 0.79), while Pb also showed strong correlations with Zn (*r* = 0.86) and Hg (*r* = 0.77). In addition, Zn and Hg were positively correlated (*r* = 0.75) (Fig. 2). Both SOM and CEC were significantly associated with the accumulation of several heavy metals. SOM was strongly correlated with Cu (*r* = 0.75) and moderately with Cr (*r* = 0.61), As (*r* = 0.54), Mn (*r* = 0.57), and Pb (*r* = 0.57). Similarly, CEC showed strong correlations with Cu (*r* = 0.71) and Cr (*r* = 0.63), and a moderate correlation with As (*r* = 0.57). These results underscore the critical roles of SOM and exchangeable sites in influencing the retention, mobility, and potential bioavailability of heavy metals in the soil environment (Fig. 2).

### Geo-accumulation index (I_geo_) of heavy metals

**Fig. 3:**
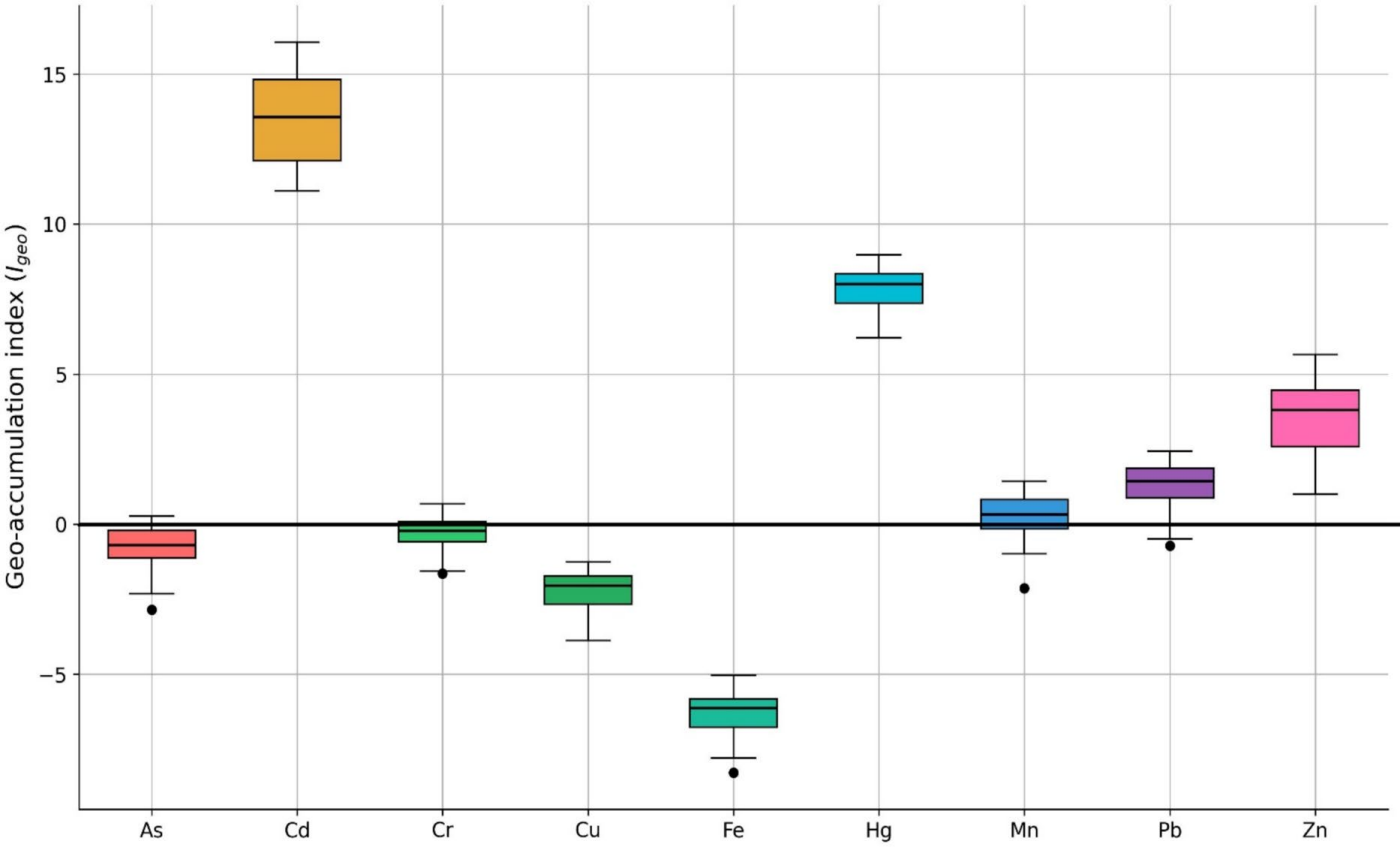
Geo-accumulation index (*I*_*geo*_) for heavy metals in soil samples. Values were calculated using the mean concentrations of replicate samples (n = 60)

The *I*_*geo*_ was employed to assess the degree of heavy metal contamination in the soil samples. The *I*_*geo*_ values for nine metals were calculated and are presented in Fig. 3. As, Cr, Cu, Mn, and Fe generally exhibited *I*_*geo*_ values ≤ 0, classifying these elements as uncontaminated, which suggests that their concentrations were close to or below regional background levels. Occasional light to moderate contamination (0 < *I*_*geo*_ ≤ 1) was observed for As at 36 out of 60 sampling sites (60%), whereas Cr and Mn did not exhibit values within this range. However, these occurrences were spatially inconsistent and did not represent a dominant contamination signal. In contrast, Pb showed predominantly light to moderate to moderate contamination (0 < *I*_*geo*_ ≤ 2), with some sites (e.g., R7, R11, R16, and R17) reaching moderate to heavy contamination (2 < *I*_*geo*_ ≤ 3), indicating localized Pb enrichment relative to background values. Zn displayed consistently elevated *I*_*geo*_ values, ranging from moderate to heavy contamination (2 < *I*_*geo*_ ≤ 4) to extremely serious contamination (*I*_*geo*_ ≥ 5), highlighting substantial Zn accumulation at several sites (e.g., R7, R11, R16–R19). Notably, Hg and Cd exhibited uniformly high *I*_*geo*_ values across all sampling locations and depths, with Hg consistently classified as extremely serious contamination (*I*_*geo*_ ≥ 5) and Cd presenting the highest *I*_*geo*_ values among all metals, clearly indicating severe and widespread contamination throughout the study area.

### Pollution load index (PLI)

**Fig. 4:**
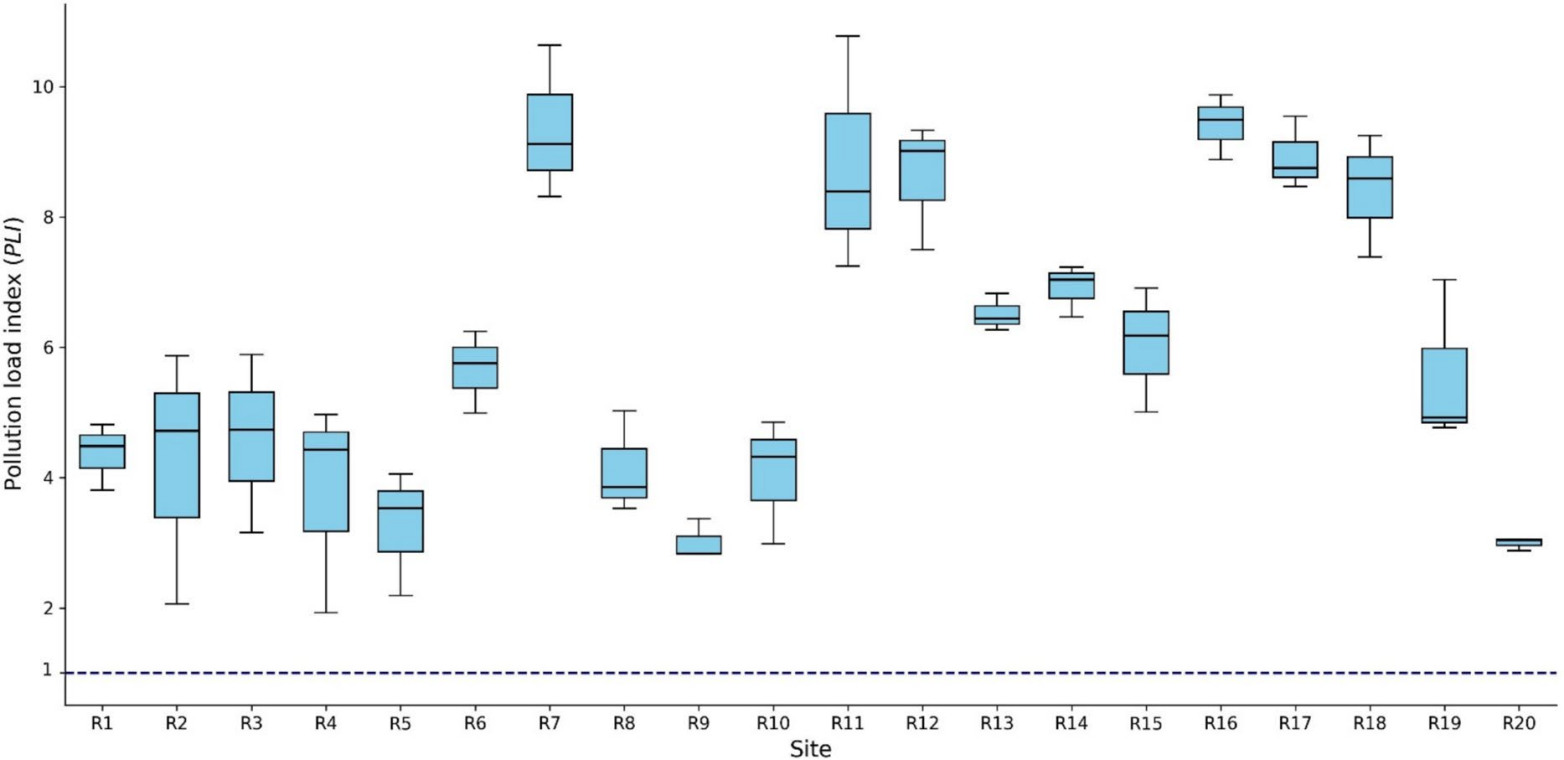
Pollution Load Index (*PLI*) for soil samples across different sites

The *PLI* was calculated for each sampling site to provide an integrated measure of heavy metal contamination (Fig. 4). All calculated *PLI* values were greater than 1, ranging from approximately 1.93 to 10.77, thereby classifying every site as polluted relative to background conditions. Lower *PLI* values (approximately 1.9–3.5) were observed at several locations, such as R4, R9, and R20, indicating comparatively lower but still polluted conditions. In contrast, markedly elevated *PLI* values were recorded at multiple sites, particularly R7, R11, R12, R16, R17, and R18, where *PLI* values frequently exceeded 8 and reached a maximum of 10.77 at R11, reflecting severe overall metal pollution. Variability in *PLI* values among sites and sampling positions suggests spatial heterogeneity in contamination intensity; however, the consistently high *PLI* values across the study area indicate a wide pollution signal rather than isolated contamination hotspots.

### Metal transfer factors (TF) in the soil–rice system

#### Transfer from soil to root (TF_soil-root_)

**Fig. 5:**
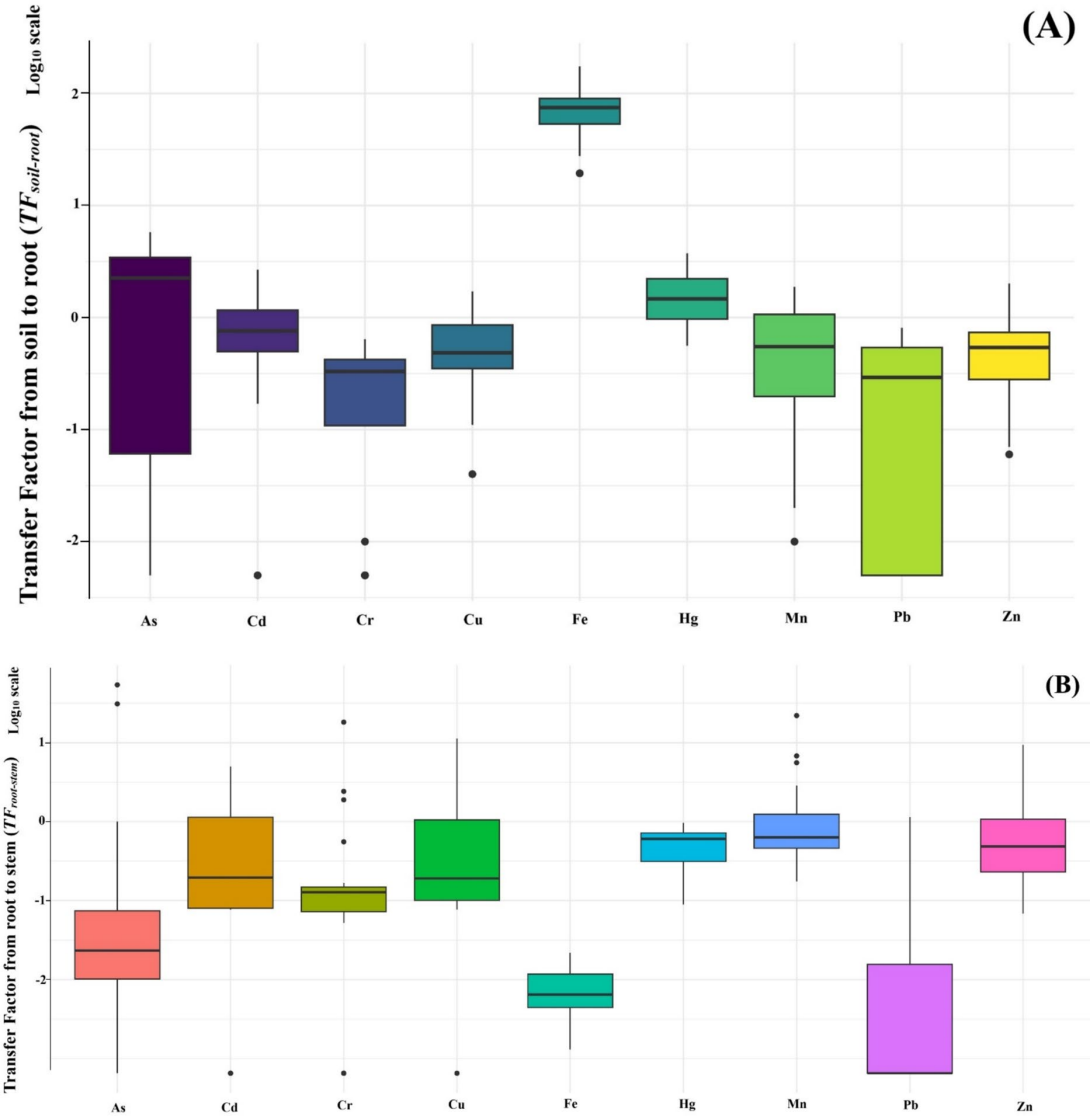

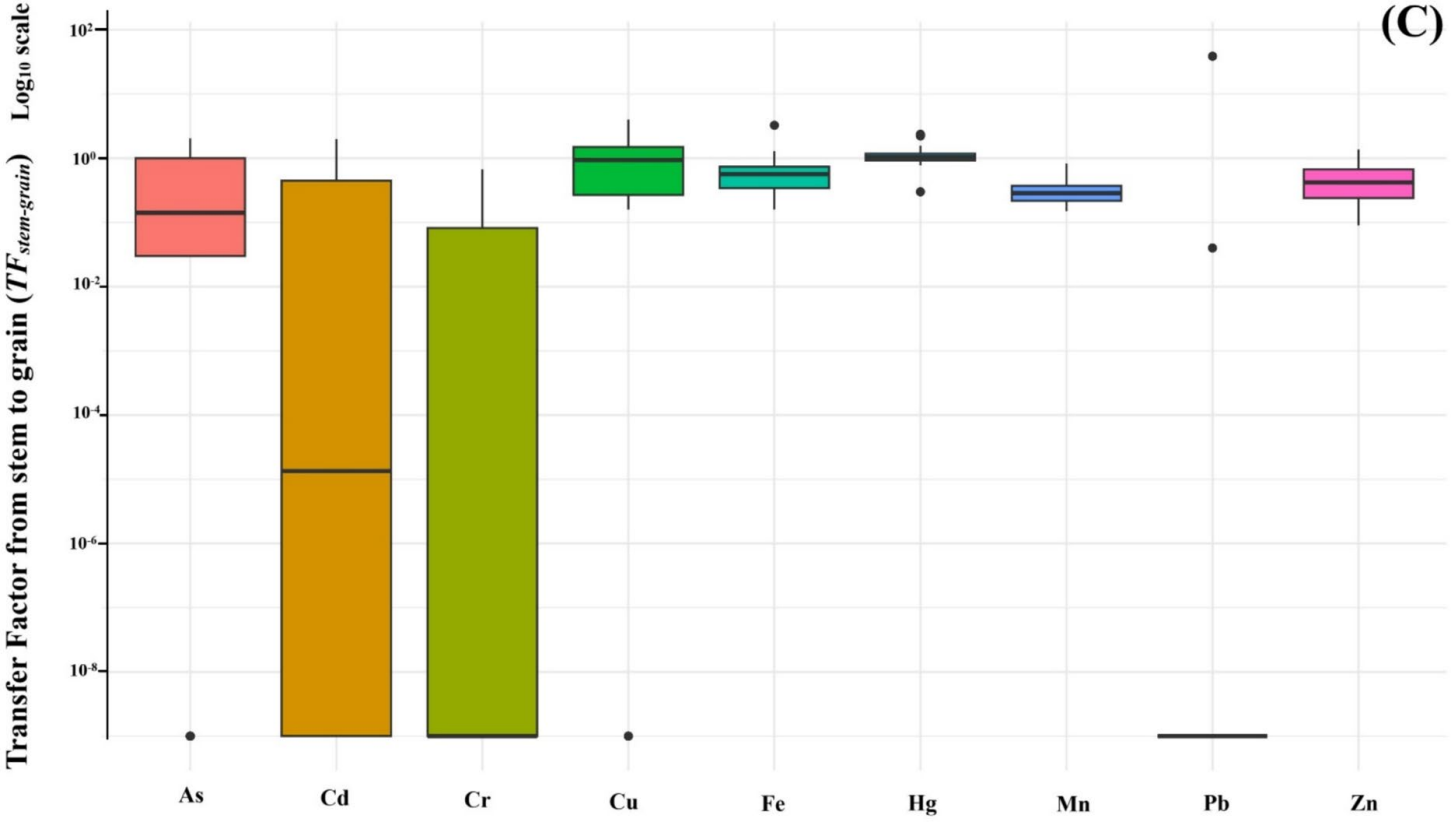
Metal transfer factors in the soil-rice system. **A** TF from soil to root, **B** TF from root to stem, and **C** TF from stem to grain

To assess the uptake potential of heavy metals by rice plants, the transfer factor from soil to root (*TF*_*soil-root*_) was calculated. Results, presented as log₁₀-transformed values, reveal notable variation among metals in their bioaccumulation potential (Fig. 5A). Fe exhibited the highest accumulation, with a median log_10_ TF of approximately 1.9, corresponding to a TF of ~ 80. Fe transfer values ranged from 19.36 to 173.81 across all sites, indicating consistent and strong uptake into rice roots. As also showed substantial accumulation, with a median log₁₀ TF of ~ 0.5 (TF > 1), and a maximum of 5.76 recorded at site R18. Hg followed a similar trend, with a median log₁₀ TF slightly above 0, suggesting modest but consistent uptake. In contrast, metals such as Cd, Cu, Mn, and Zn had median log_10_ TF values between − 0.5 and 0, indicating limited root accumulation. The lowest transfer efficiency was observed for Cr and Pb, with median log₁₀ TF values around − 0.6 and − 0.7, respectively. In some locations, Pb showed TF values of zero, highlighting its extremely low mobility into plant roots. Overall, the relative uptake efficiency from soil to root follows the descending order: Fe» As > Hg > Cd > Zn > Cu > Mn > Cr > Pb, emphasizing Fe as an essential nutrient actively absorbed by roots and As as a non-essential element with high root accumulation potential.

#### Transfer from root to stem (TF_root-stem_)

The internal translocation of metals from roots to aerial parts was evaluated using the root-to-stem transfer factor (TF_(root-stem)_). Figure 5B, based on log₁₀-transformed values, reveals distinct patterns of metal mobility. Fe and Pb were the least mobile, with median log₁₀ TFs of approximately − 2.2 and − 2.8, respectively, indicating strong retention in roots. As also showed poor mobility (median log₁₀ TF ~ − 1.5), although an extreme outlier at site R4 (TF = 54) suggests site-specific conditions may influence its movement. Moderate to low translocation was observed for Cd, Cr, and Cu, with median log₁₀ TFs between − 0.7 and − 0.9. However, high TFs were recorded in specific sites (e.g., Cd = 31 at R6; Cr = 5 at R3, R6, R7; Cu = 11.3 at R6), pointing to localized factors enhancing metal mobility. Conversely, Mn, Hg, and Zn displayed relatively higher internal mobility, with median log₁₀ TFs around − 0.4 to − 0.2. For these elements, TF values > 1 were observed at several sites, suggesting a greater capacity for root-to-stem translocation. The general trend for metal mobility from root to stem was: Mn ≈ Hg ≈ Zn > Cu > Cd > Cr > As > Fe > Pb, reflecting a clear contrast between mobile micronutrients and poorly translocated toxic metals.

#### Transfer from stem to grain (TF_stem-grain_)

Metal mobility into the edible part of the plant was evaluated using *TF*_*stem-grain*_ (Fig. 5C). The results reveal considerable variability among metals in their ability to translocate from vegetative to reproductive tissues. Cu and Hg showed the highest translocation efficiencies, both with median TFs around 1.0. Fe, Zn, and Mn followed closely, with median TFs of ~ 0.8, 0.6, and 0.6, respectively. These results align with known physiological behavior, as micronutrients like Cu and Zn are efficiently mobilized to the grain. Some Cu and Zn TFs ranged from 1.1 to 2.5. As exhibited moderate but variable translocation (median ~ 0.2, range 0.02–1.0), reflecting the complex behavior of As in rice transport systems. In contrast, Cd, Cr, and Pb exhibited extremely limited translocation. Median TFs for Cd and Cr were approximately 3 × 10⁻^5^ and 3 × 10⁻^2^, respectively, while Pb showed the lowest mobility with a median TF near 1 × 10^−6^, indicating near-total retention in the vegetative tissues. The TF variability, particularly for Cd and Cr, spanned several orders of magnitude, pointing to site-specific environmental or plant-based influences. Notable outliers included unexpectedly high Pb translocation in one site, and a low Cu TF in another, underscoring the importance of localized factors. The overall translocation trend from stem to grain was: Cu ≈ Hg > Fe ≈ Zn ≈ Mn > As >  > Cr > Cd >  > Pb, emphasizing the higher mobility of essential nutrients and the restricted movement of toxic metals like Pb and Cd.

### Health risk assessment from rice consumption

**Fig. 6:**
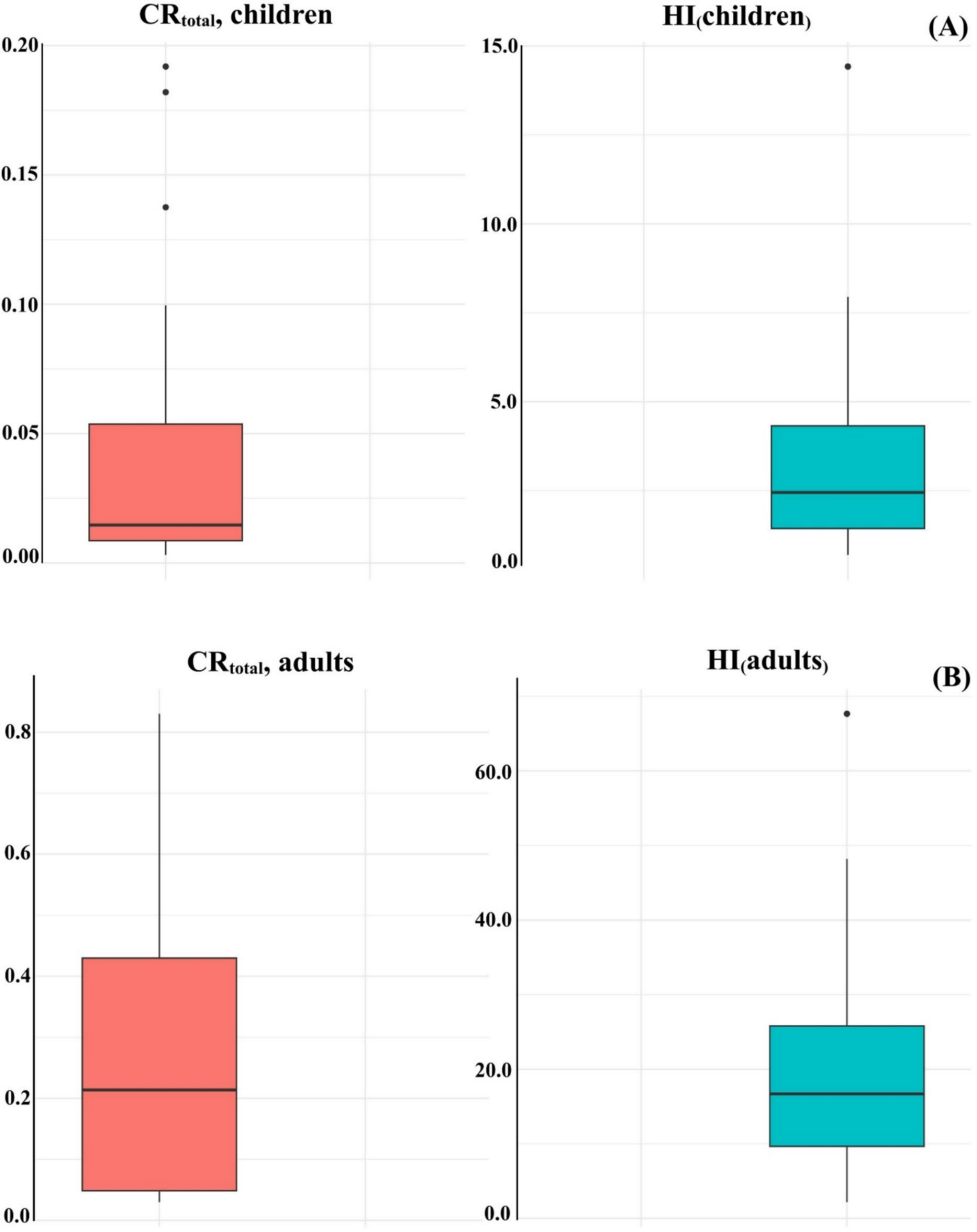
Health risk assessment for rice consumption. **A** Total carcinogenic risk (CR_total_) and Hazard Index (HI) for children. **B** Total carcinogenic risk (CR_total_) and Hazard Index (HI) for adults

The health risks to children from rice consumption were evaluated using two indicators: the non-carcinogenic hazard index (HI₍children₎) and the total carcinogenic risk (CRₜₒₜₐₗ, children) (Fig. 6A). The HI₍children₎, calculated as the sum of hazard quotients for multiple heavy metals, reflects the potential for non-carcinogenic adverse effects, with values exceeding 1 indicating potential health concern. In this study, HI₍children₎ values ranged from 0.70 (Site R18) to 14.42 (Site R5), with the majority of sites (17 out of 20) exceeding the safety threshold, indicating widespread non-carcinogenic risk. The carcinogenic risk (CRₜₒₜₐₗ, children) estimates the lifetime cancer risk associated with exposure to carcinogenic metals. Acceptable risk levels typically fall within the range of 1 × 10^−6^ to 1 × 10⁻^4^, while values exceeding 1 × 10⁻^4^ indicate significant concern. In this study, CRₜₒₜₐₗ values ranged from 3.10 × 10⁻^3^ (Site R19) to 1.92 × 10⁻^1^ (Site R3), with all sites exceeding the acceptable threshold. The highest carcinogenic risks were observed at Sites R2, R3, and R9. Overall, these results indicate a substantial and widespread carcinogenic risk to children in the study area.

Health risks to adults consuming rice from the same locations were assessed using the hazard index (HI₍adults₎) and total carcinogenic risk (CRₜₒₜₐₗ, adults), revealing similarly concerning trends (Fig. 6B). The HI₍adults₎ values ranged from 2.18 (Site R1) to 67.7 (Site R14), with all sampling sites exceeding the acceptable safety threshold (HI = 1), indicating widespread non-carcinogenic risk. The highest HI values were observed at Sites R14 (67.7), R15 (48.2), and R11 (42.4), identifying these locations as critical hotspots. Similarly, the CRₜₒₜₐₗ values for adults ranged from 3.00 × 10⁻^2^ (Site R19) to 8.30 × 10⁻^1^ (Site R1), with all sites exceeding the acceptable risk level (1 × 10⁻^4^). The highest carcinogenic risks were observed at Sites R1, R16, and R17. Even the lowest CRₜₒₜₐₗ values indicate a substantial carcinogenic risk, highlighting the urgent need for mitigation measures across the study area.

## Discussion

### Heavy metal contamination in the soil–rice system

This study demonstrates pronounced heavy metal accumulation in the soil–rice system, reflecting the long-term legacy and continuing influence of zinc mining activities in the Mae Sot area. Although the total concentrations of all measured metals in soil remained below the national regulatory limits established by the Pollution Control Department (2021), the *I*_*geo*_ and *PLI* revealed substantial enrichment relative to natural background levels. In particular, Cd and Hg exhibited extremely serious contamination (*I*_*geo*_ ≥ 5) at all sampling sites, while Zn similarly reached this category at several locations, highlighting severe anthropogenic inputs that are not fully captured by regulatory threshold-based assessments. These findings emphasize that compliance with soil quality standards does not necessarily indicate the absence of environmental contamination risks.

The widespread elevation of *PLI* values (> 1) across the entire study area further confirms the presence of widespread multi-metal pollution rather than isolated contamination hotspots. Sites with especially high *PLI* values, including R7, R11, R12, R16, R17, and R18, reflect areas where cumulative metal inputs are particularly pronounced. Such spatial patterns likely result from the combined effects of historical mine tailings, smelter-related atmospheric deposition, irrigation water contaminated by upstream mining residues, and sediment transport and redistribution along Mae Tao Creek. The consistency of elevated Cd and Hg contamination across all sites suggests persistent and long-term pollution sources, whereas the spatial variability observed for Zn and Pb indicates additional localized enrichment processes. Together, the *I*_*geo*_ and *PLI* results highlight the importance of integrated contamination indices for identifying chronic and cumulative pollution in mining-affected agroecosystems, even where bulk soil concentrations remain within regulatory limits.

### Uptake and translocation dynamics of heavy metals

The transfer factor analysis across soil–root–stem–grain compartments provides critical insights into metal mobility and plant uptake behavior. Fe demonstrated an extraordinarily high *TF*_*soil-root*_, consistent with its role as an essential micronutrient and its high availability under flooded conditions (Wang et al., 2022). As and Hg, both toxic non-essential elements, also showed relatively high root accumulation (TF > 1), suggesting strong bioavailability, likely influenced by redox-induced mobilization and soil texture characteristics (Gil-Díaz et al., 2021; Rinklebe et al., 2020). In contrast, Cd, Cu, Mn, and Zn exhibited limited root accumulation (TF < 1), likely due to plant homeostatic regulation, competitive ion uptake or sorption to soil organic matter and clay minerals. Pb and Cr showed minimal uptake into roots, consistent with their known strong affinity for soil particles and poor solubility under neutral to alkaline pH conditions—conditions that dominated the studied soils (Nawrot et al., 2023; Rooney et al., 2007).

Translocation from root to stem was notably restricted for most metals, especially Fe, Pb, and As, whose mobility was hindered by sequestration in root tissues. However, Mn, Hg, and Zn demonstrated comparatively greater mobility, suggesting a capacity for internal redistribution, particularly under conditions of micronutrient demand. This aligns with earlier reports that Zn and Mn exhibit higher phloem mobility, especially in younger tissues (Jiang et al., 2025; Page & Feller, 2015).

From the stem to grain, Cu and Hg had the highest TFs, with Fe, Zn, and Mn also showing moderate translocation. In contrast, Cd, Cr, and Pb showed extremely low grain accumulation, yet their concentrations in the grain still exceeded food safety thresholds—suggesting that even limited translocation can result in hazardous accumulation when soil concentrations are elevated or plant barriers are compromised.

### Influence of soil properties on metal behavior

Correlational analysis highlights the key role SOM and CEC in regulating heavy metal retention and mobility. SOM was significantly correlated with the accumulation of Cu, Cr, As, and Pb, indicating its function in complexation and surface adsorption processes that influence metal bioavailability (Li et al., 2024; Yu et al., 2023). Functional groups in organic matter, such as carboxyl (–COOH) and hydroxyl (–OH), can form stable organo-metal complexes, thereby enhancing metal retention (Ferreira et al., 2025). At the same time, dissolved organic carbon (DOC) fractions may increase the mobility of certain metals, such as As, through the formation of soluble complexes, highlighting the dynamic role of SOM in both immobilization and mobilization processes (Lu et al., 2007).

CEC showed strong positive correlations with Cu, Cr, As and Mn, reflecting the abundance of negatively charged exchange sites on clay minerals and organic matter (Agarwal et al., 2025; Stathi et al., 2010). These sites facilitate the adsorption of metal cations via electrostatic attraction and cation exchange processes, thereby reducing their mobility in soil solution (Thompson & Goyne, 2012). However, exchangeable metal fractions may remain partially bioavailable, particularly under conditions where competing cations are present. In the alkaline soils of the study area, high concentrations of Ca^2^⁺ and Mg^2^⁺ can compete with trace metals for adsorption sites, influencing both retention and plant uptake (Meers et al., 2009).

Additionally, soil texture emerged as a key determinant of metal dynamics. Sandy soils were associated with lower SOM, total N, and metal concentrations—particularly As and Cu—suggesting reduced retention capacity and higher leaching potential (Huang & Hartemink, 2020; Matichenkov et al., 2020). Conversely, clay-rich soils were more effective at retaining metals, likely through higher specific surface area and adsorption capacity (Agarwal et al., 2025; Otunola & Ololade, 2020).

### Human health risks from rice consumption

One of the most significant findings of this study is the elevated health risk associated with rice consumption for both children and adults. For children, HI values ranged from 0.70 to 14.42, with the majority of sites exceeding the safety threshold (HI = 1), indicating potential non-carcinogenic health risks. The CRₜₒₜₐₗ ranged from 3.10 × 10⁻^3^ to 1.92 × 10⁻^1^, substantially exceeding the acceptable limit of 1 × 10⁻^4^. For adults, a similar pattern was observed, with HI values ranging from 2.18 to 67.7, and CRₜₒₜₐₗ values ranging from 3.00 × 10⁻^2^ to 8.30 × 10⁻^1^. In all cases, carcinogenic risks exceeded acceptable thresholds by several orders of magnitude, indicating widespread and potentially serious long-term health implications for local populations.

Sites R3, R5, R7, R11, R14, R16, and R17 repeatedly emerged as risk hotspots, aligning with their elevated *PLI* and grain metal concentrations. These findings are consistent with prior studies from Thailand and other mining regions that report severe health outcomes—including renal dysfunction, neurotoxicity, and elevated cancer incidence—linked to rice-based exposure to Cd, As, and Pb (Chaiwonga et al., 2013; Songprasert et al., 2015). Moreover, high HI and CR values at certain sites reflect the cumulative effect of elevated grain metal concentrations combined with conservative exposure assumptions, and should therefore be interpreted as indicators of relative risk severity and hotspot identification rather than precise individual risk estimates.

In this study, human health risk assessment was conducted using total concentrations of As, Cd, Cr, Cu, Mn, Pb, Zn, Hg, and Fe, following a conservative screening-level approach commonly applied in preliminary risk evaluations. This approach may therefore lead to an overestimation of actual human exposure. While this assumption is generally acceptable for toxic elements such as As, Cd, Pb, Hg, and Cr, it is particularly relevant for essential elements including Cu, Fe, Zn, and Mn, which are often present in mineral-bound or strongly adsorbed forms and are subject to biological regulation. Consequently, the estimated health risks should be interpreted as worst-case scenarios rather than realistic exposure levels. The present findings demonstrate that compliance with bulk soil quality standards alone may be insufficient to ensure food safety in mining-affected agroecosystems. Although total metal concentrations in soil may fall within regulatory thresholds, metal transfer to rice grains can still occur due to bioavailability, soil physicochemical conditions, and plant-specific uptake mechanisms. Therefore, regulatory frameworks should move beyond total soil concentration criteria and adopt more integrated assessment approaches that incorporate bioavailable metal fractions, soil properties (e.g., pH, SOM, and CEC), and crop-specific transfer factors. In addition, routine monitoring programs in mining-impacted agricultural areas should include simultaneous assessment of soil, irrigation water, and edible crop tissues, particularly rice grains, to better capture human exposure risks. Spatially targeted surveillance in high-risk areas near mining operations and the development of site-specific management thresholds may further improve risk mitigation and food safety protection in rice-based agroecosystems.

## Conclusion

This study set out to evaluate heavy metal contamination in a Zn mining–affected soil–rice system, identify soil properties influencing metal mobility and uptake, and assess the associated human health risks. The *I*_*geo*_ revealed extremely serious contamination by Cd and Hg across all sites, with Zn also reaching extremely serious levels at several locations, while As, Cr, Mn, Cu, and Fe were largely within background to slightly contaminated ranges. Consistently elevated *PLI* values (> 1) at all sampling sites confirmed widespread multi-metal pollution throughout the study area. Soil properties, particularly SOM, CEC, and texture, were shown to play critical roles in regulating metal retention and transfer, with sandy soils exhibiting reduced capacity to retain metals and clay-rich soils enhancing accumulation. Transfer factor analysis revealed that while essential elements (e.g., Cu, Fe, Zn, Mn) showed higher grain translocation, toxic metals such as As and Hg accumulated predominantly in roots. Notably, even the limited mobility of Pb and Cd resulted in grain concentrations exceeding food safety limits established by the Ministry of Public Health, Thailand (2020). Health risk assessments demonstrated that rice consumption poses severe risks to both children and adults, with hazard indices and cancer risk values far surpassing acceptable thresholds. These results strongly support the conclusion that rice cultivated in mining-affected areas represents a major exposure pathway for toxic metals, posing significant risks to food safety and public health. To mitigate these risks, targeted management strategies to reduce metal bioavailability, and regular monitoring of soil and rice grain quality should be implemented.

## Supplementary Information

Below is the link to the electronic supplementary material.

Supplementary file1 (DOCX 15 KB)

## Data Availability

All data generated or analysed during this study are included in this published article.
