# Supplementary material for: Heavy metal transfer and health risk assessment in an abandoned zinc mining–affected soil–rice system in western Thailand
Source: Environ Geochem Health. 2026 May 27;48(8):375. doi: 10.1007/s10653-026-03253-1 (PMC13216163; doi:10.1007/s10653-026-03253-1)
Supplement: Supplementary file 1 — Supplementary file1 (DOCX 15 KB) [file 10653_2026_3253_MOESM1_ESM.docx]

**Supplementary Material**

**Table S1.** Oral reference doses (RfD) and slope factor for carcinogenic (CSF) of heavy metals.

| **Elements** | **Adult** | | **Children** | |
| --- | --- | --- | --- | --- |
|  | **RfD** (mg/kg/day) | **CSF** (mg/kg/day) | **RfD** (mg/kg/day) | **CSF** (mg/kg/day) |
| As | 3.00 × 10^-4a^ | 1.50 × 10^0b^ | 3.00 × 10^-4a^ | 1.50 × 10^0b^ |
| Hg | 3.00 × 10^-4a^ | - | 3.00 × 10^-4a^ | - |
| Pb | 1.40 × 10^-3a^ | 0.0085^b^ | 1.40 × 10^-3a^ | 0.0085^b^ |
| Cd | 5.00 × 10^-4a^ | 15^b^ | 5.00 × 10^-4a^ | 15^b^ |
| Cr | 3.00 × 10^-4a^ | 0.50 × 10^0a^ | 3.00 × 10^-4a^ | 0.50 × 10^0a^ |
| Cu | 4.00 × 10^-2c^ | - | 4.00 × 10^-2c^ | - |
| Mn | 0.14^d^ | - | 0.14^d^ | - |
| Zn | 0.30^d^ | - | 0.30^d^ | - |
| Fe | 0.70^d^ | - | 0.70^d^ | - |

^a^ Wahyuningsih, N.E., Setiawan, H., Nabiha, P.I., Kartasurya, M.I., Azam, M. 2023. Heavy

Metals Contamination of Local and Imported Rice in Semarang, Central Java, Indonesia. Journal of Ecological Engineering, 24(7), 49–60.

^b^ USEPA. 2005. Guidelines for carcinogen risk assessment; EPA/630/P03/001F. Risk

Assessment Forum, Washington, DC, USA.

^c^ Javed, M., Usmani, N. 2016. Accumulation of heavy metals and human health risk

assessment via the consumption of freshwater fish *Mastacembelus armatus* inhabiting, thermal power plant effluent loaded canal. Javed and Usmani SpringerPlus, 5, 776.

^d^ US EPA (United States Environmental Protection Agency). 2000. Handbook for non-cancer

health effects evaluation. US Environmental Protection Agency, Washington (DC).
